# *Helicobacter pylori* infection and association with chronic diseases: A focus on cardiovascular disease, MASLD, and type 2 diabetes

**DOI:** 10.1016/j.metop.2025.100385

**Published:** 2025-08-11

**Authors:** Navid Maleki, Alireza Mohammadzadeh, Jalal Mardaneh, Hossein Pazoki, Elyas Nattagh-Eshtivani

**Affiliations:** a Student Research Committee, Infectious Diseases Research Center, Gonabad University of Medical Sciences, Gonabad, Iran; b Department of Microbiology, Faculty of Medicine, Infectious Diseases Research Center, Gonabad University of Medical Sciences, Gonabad, Iran; c Department of Nutrition and Biochemistry, Faculty of Medicine, Infectious Diseases Research Center, Gonabad University of Medical Sciences, Gonabad, Iran

**Keywords:** *Helicobacter pylori*, Cardiovascular disease, Metabolic dysfunction-associated steatotic liver disease, Diabetes mellitus

## Abstract

*Helicobacter pylori* (*H. pylori*) infection is a globally prevalent gastrointestinal pathogen increasingly linked to various extra-gastric non-communicable diseases (NCDs). This review addresses the guiding question: What epidemiological and mechanistic links explain the association between H. pylori infection and chronic conditions such as cardiovascular disease (CVD), metabolic dysfunction-associated steatotic liver disease (MASLD), and type 2 diabetes mellitus (T2DM)?

The manuscript synthesizes evidence from epidemiological studies and mechanistic research. In CVD, *H. pylori* exacerbates chronic vascular inflammation, endothelial dysfunction, and autoimmune-like responses such as molecular mimicry. In MASLD, *H. pylori* induces insulin resistance (IR), hepatic inflammation, and microbiota-mediated liver injury, although findings remain inconclusive across populations. For T2DM, multiple pathways including NLRP3 inflammasome activation, hormonal imbalances (e.g., ghrelin, leptin), and immune-genetic interactions involving TLR4 and SOCS3 suggest a role for *H. pylori* in metabolic dysregulation and impaired glycemic control. While researchers have not yet fully elucidated causality, these findings indicate *H. pylori* as a potential modifiable risk factor for NCDs. Future longitudinal and interventional studies are warranted to determine whether eradication of *H. pylori* can mitigate chronic disease.

## 1 Introduction

According to the Centers for Disease Control and Prevention (CDC), chronic illnesses, sometimes referred to as non-communicable diseases (NCDs), are ailments that persist for a year or more and restrict everyday activities or require continuous medical care. The World Health Organization (WHO) estimates that NCDs cause 71 % of all deaths worldwide. NCDs are a prominent concern underlined in the 2030 sustainable development goals (SDGs), which seek to reduce all deaths from NCDs by one-third by 2030 [1].

Based on studies, over the past three decades, global disease burden has moved from communicable to NCDs [2]. According to the worldwide disease burden, the four leading causes of mortality from NCDs are cardiovascular disease (41 %), cancer (25 %), chronic respiratory diseases (10 %), and diabetes (4 %) [3] However, NCDs can influence health in other regions as well, such as the liver (MASLD) [4]. Also, human papillomavirus (HPV) and *H. pylori*, which cause stomach adenocarcinoma and cervical cancer, are examples of microbial infections linked to chronic non-communicable conditions [5].

*H. pylori* is a gram-negative, microaerophilic, helical bacillus [6] and is one of the most prevalent infections in the world, leading to gastric cancer [7]. The world's gastric cancer death rate reaches 720,000 cases per year, and it is the third most common cause of cancer death [8]. Also, there is a wide range in the prevalence of this bacterium based on the geographic location, ranging from 25 to 50 % in developed countries to over 80 % in underdeveloped countries [9]. We also see a decrease in *H. pylori* prevalence in more prosperous countries, largely due to improved hygiene, sanitation, and living conditions, which reduce the likelihood of early childhood transmission, the primary mode of infection.

Infection with *H. pylori* is connected with numerous gastrointestinal diseases, such as gastric inflammation, peptic ulcers, gastric cancer, and mucosa-associated lymphoid tissue lymphomas [[10], [11], [12], [13]], *H. pylori* also plays an important part in extra-gastric diseases, as Mendel et al. first reported in 1994 [14]. Since then, many studies have investigated the role of *H. pylori* in extra-gastric diseases.

The majority of diabetes patients referred to diabetes clinics report digestive symptoms like dysphagia, reflux, constipation, abdominal pain, vomiting and diarrhea [15], and up to 75 % of these patients will experience significant digestive symptoms [16,17]. The digestive system can be affected almost entirely by diabetes considering *H. pylori* infection is mostly responsible for digestive symptoms, many articles have investigated its association with diabetes, and for example a study conducted in 2016 found that diabetics with *H. pylori* infection needed more intensive treatment to achieve comparable glucose levels. Also, eradicating *H. pylori* improves the control of blood sugar by reducing hemoglobin A1C (HbA1C) [18]. Also, another study released in 2022 found that *H. pylori* infection increases type 2 diabetes risk (T2DM) [19]. A meta-analysis study conducted in 2024 also showed a significant association between *H. pylori* infection and diabetes duration as well as HbA1c levels [20].

Metabolic dysfunction-associated steatotic liver disease (MASLD) is a complicated condition that is affected by numerous factors, such as genetics and environmental [21,22]. Studies have shown that MASLD is influenced by the microbiota in the gastrointestinal tract [23,24], Consequently, articles have been published investigating the role *H. pylori* plays in MASLD, in the study conducted by Abo-Amer et al., *H. pylori* was found to be an independent risk factor for MASLD [25]. Also a recent bidirectional meta-analyses involving 175,575 individuals showed evidence of a reciprocal association between *H. pylori* infection and MASLD [26]. In contrast to the previous study, the results of Fan's study suggest that MASLD does not show a link with *H. pylori* infection in healthy individuals [27]. Similarly, another cohort study conducted on 2063 adults found no evidence of an association between *H. pylori* infection and MASLD [28].

The term CVD refers to several conditions that affect the heart, including hypertension, coronary artery disease (CAD), and atherosclerosis [29,30]. The investigations now suggests that virus and bacterial pathogens, such as *H. pylori*, are factors contributing to coronary heart disease (CHD) development [31]. In one study conducted in 2022 by Pina Dore et al. , *H. pylori* infection, especially long-lasting infection, was identified as an independent risk factor for hypertension and carotid plaques [32]. Additionally, a 2021 study suggested that *H. pylori* infection contributes to the development of atherosclerosis through non-traditional mechanisms, such as immune-mediated inflammation and endothelial dysfunction, rather than through classical risk factors alone [33]. Recent studies have also provided much evidence for the association between *H. pylori* and CVD. For example, a comprehensive meta-analysis in 2025 showed a strong association between *H. pylori* infection and the risk of CVD problems, as well as a high prevalence of *H. pylori* infection among CVD. Also, a meta-analysis that included 41 cohort studies and approximately 230,288 participants demonstrated that *H. pylori* infection increases the risk of CVD by about 10 % [34].

Given the rising global burden of NCDs and the high prevalence of *H. pylori* infection, this review aims to synthesize the current evidence supporting a potential association between *H. pylori* infection and major chronic conditions, specifically CVD, MASLD, and T2DM. The primary objective is to assess both epidemiological data and proposed biological mechanisms such as chronic inflammation, IR, immune modulation, and microbial alterations that underlie these associations. Additionally, the review explores the influence of host genetic factors and microbial dysbiosis in modulating disease outcomes. By critically analyzing the available literature, this review seeks to clarify the potential extra-gastric impacts of *H. pylori*, identify existing knowledge gaps, and offer directions for future research and public health strategies.

## 2 Role of host genetic polymorphisms in H. pylori susceptibility and disease progression

Recent research has highlighted the critical role of host genetic polymorphisms in modulating susceptibility to *H. pylori* infection and influencing disease outcomes. A large-scale study demonstrated that individuals carrying the TLR10 rs10004195 AA genotype have a significantly increased risk of *H. pylori* infection, implicating innate immune recognition pathways in host susceptibility [35]. Additionally, polymorphisms in cytokine genes such as IL-1B-31C/T, IL-1B-511C/T, and IL-8-251 T/A affect both the risk of infection and the severity of associated diseases, including gastric inflammation and carcinogenesis [36]. Beyond infection risk, genetic variations also impact treatment response; for instance, certain polymorphisms can alter the efficacy of eradication regimens and increase the likelihood of antibiotic resistance [37,38]. These findings underscore the importance of considering genetic background in both the clinical management of *H. pylori* infection and the development of personalized therapeutic strategies.

## 3 H. pylori and cardiovascular diseases

During the 20th century, the prevalence and death rates from communicable diseases decreased, while NCDs increased. Meanwhile, CVDs cause the highest mortality and disability among NCDs [39]. The mortality rate from CVDs has decreased greatly in recent years, which many factors contribute to, such as healthier diets, lower smoking rates, better management of conditions such as hypertension, and improved secondary prevention [40]. Despite the difficulty in determining the exact mechanism linking *H. pylori* and heart disease, this topic has attracted many scientists in recent years [41].

### 3.1 Epidemiological associations

Azarkar et al. conducted a case-control study involving 151 subjects, and identified an association between *H. pylori* infection and myocardial infarction. Furthermore, *H. pylori* contributes to atherosclerosis or exacerbate other risk factors [42]. In a study involving 170 patients with CHD, Matsuiak et al. found an increased risk of vascular pathologies in patients exposed to *H. pylori*. They also observed higher levels of lipopolysaccharide-binding protein (LBP) in the CHD group compared to those without CHD. This finding highlights a possible link between *H. pylori* and CHD [43]. Eskandarian et al. conducted a prospective cohort study on 433 individuals, highlighting that *H. pylori* infection may impact the early prognosis of CAD patients. However, the study found a non-significant association between *H. pylori* infection and hypertension, diabetes mellitus (DM), hyperlipidemia, smoking, or a family history of CVDs [44].

Since hypertension is considered a major risk factor, many studies have examined its relationship with *H. pylori*. Xiong et al. designed a study in 2008 involving 17,100 participants that was published in 2020. According to this study, hypertension and *H. pylori* infection show a clear correlation. They found a clear correlation between hypertension and *H. pylori* infection. Their analysis further revealed an independent association between *H. pylori* infection and elevated diastolic blood pressure (DBP), but not with systolic blood pressure (SBP), pulse pressure (PP), or mean arterial pressure (MAP). Additionally, *H. pylori* infection reduces with high-density lipoprotein cholesterol (HDL-C) and increases total cholesterol (TC), low-density lipoprotein cholesterol (LDL-C), and fasting plasma glucose (FPG) levels [45]. Additionally, a community-based case-control study by Hasan et al. demonstrated a relationship between *H. pylori* infection and both blood pressure and mean arterial pressure (MAP), suggesting that treatment of the infection helps prevent hypertension [46]. Further, Choi et al. designed a study on 2251 subjects to determine the relationship between *H. pylori* and arterial stiffness. This study demonstrated an association between chronic *H. pylori* infection and arterial stiffness in asymptomatic individuals [47]. Their results further supported the idea that persistent *H. pylori* infection can significantly impact vascular function [47].

Recent high-quality meta-analyses provide robust evidence linking *H. pylori* infection with an increased risk of CVD. A comprehensive 2025 meta-analysis of 87 studies found that the prevalence of *H. pylori* infection among patients with vascular diseases was 56.7 %, with CAD being the most common complication (31.07 %) [48]. The study concluded that *H. pylori* infection increases the risk of CVD and suggested screening for *H. pylori* in high-risk patients. Additionally, a 2023 meta-analysis of 41 cohort studies involving 230,288 participants demonstrated that *H. pylori* increases the risk of overall CVD and CHD by 10 % (RR: 1.10, 95 % CI: 1.03–1.18). The risk was even higher among patients infected with CagA-positive *H. pylori* strains (RR: 1.58, 95 % CI: 1.03–2.41) [34]. Other meta-analyses have also reported significant associations between *H. pylori* infection and acute coronary syndrome (ACS), especially in developing countries [49]. Table 1 summarizes additional studies that examine the link between *H. pylori* and CVDs. These findings underscore the importance of considering *H. pylori* as a potentially modifiable risk factor in CVD prevention and management strategies.

**Table 1:** Summary of key studies on *H. pylori* infection and cardiovascular diseases.

Author (Country)	Population (n)	Key Findings/Regional Notes	Refs.
Migneco et al. (Italy)	142	BP reduced after *H. pylori* eradication in hypertensive patients	[61]
Pina Dore et al. (Italy)	7145	Long-term *H. pylori* is an independent risk factor for hypertension & plaques	[32]
Nguefak Tali et al. (Cameroon)	363	*H. pylori* eradication linked to improved lipid profile	[62]
Zhang et al. (China)	12386	*H. pylori* linked to carotid atherosclerosis in men <50	[63]
Wang et al. (JAPAN)	58413	Eradication associated with reduced CHD incidence	[64]
Wan et al. (China)	5168	*H. pylori* Infection increases the risk of hypertension	[65]
Wang et al. (China)	585	Patients with long-standing AF had significantly higher values of *H. pylori* than those with short-standing AF and control groups	[66]
Figura et al. (Italy)	103	CagA + *H. pylori* strains linked with higher BNP levels in CHD patients	[67]
Rogha et al. (Iran)	105	No independent association between CagA + *H. pylori* and CHD severity	[68]
Rožankovic'et al. (Croatia)	64	Higher anti-CagA antibody titers in symptomatic vs. asymptomatic carotid disease	[69]
Nyberg et al. (Sweden)	119	No link between CagA + strains and abdominal aortic aneurysm rupture	[70]

*H. pylori*: *Helicobacter pylori*, CVD: Cardiovascular disease, CHD: Coronary heart disease, AF: Atrial fibrillation, BP: Blood pressure.

### 3.2 Inflammatory and immune mechanisms

**Fig. 1:**
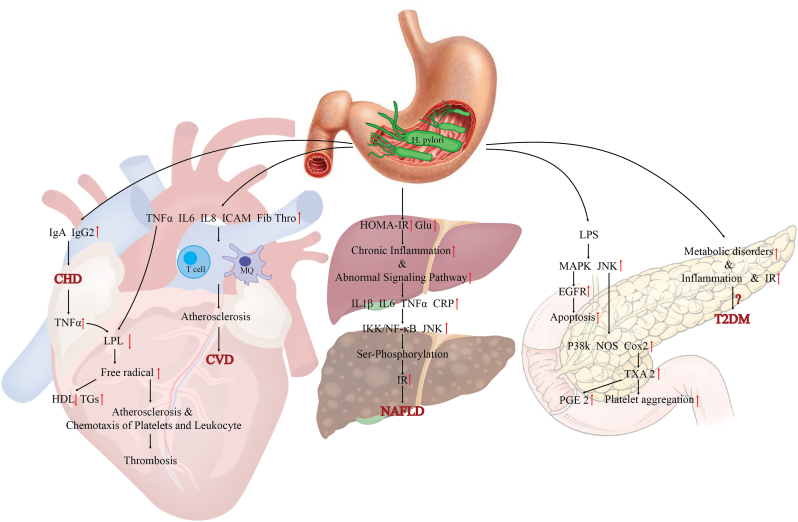
The relationship between Helicobacter pylori and chronic diseases, including cardiovascular diseases, non-alcoholic fatty liver disease, and diabetes. **Legend:** Proposed mechanisms linking *Helicobacter pylori (H. pylori)* infection to chronic metabolic diseases including cardiovascular disease (CVD), metabolic dysfunction-associated steatotic liver disease (MASLD), and type 2 diabetes mellitus (T2DM). *H. pylori* infection may contribute to the pathogenesis of chronic diseases through multiple pathways, including immune modulation (e.g., increased IgA/IgG2, TNF-α), systemic inflammation (elevated IL-6, IL-1β, CRP), oxidative stress, and dysregulation of lipid and glucose metabolism. In the heart, *H. pylori* is associated with atherosclerosis, thrombosis, and altered lipid profiles, promoting CVD and coronary heart disease (CHD). In the liver, chronic inflammation and abnormal signaling pathways (e.g., IKK/NF-κB, JNK) contribute to insulin resistance (IR) and MASLD. In the pancreas, LPS-mediated pathways activate MAPK and EGFR signaling, leading to apoptosis, metabolic disturbances, and enhanced risk of T2DM.

While the explanation for this relationship remains unclear, researchers have proposed several mechanisms. Matsioak et al., [43] found that chronic *H. pylori* infection produces higher levels of IgG2 and IGA in people with CHD than healthy subjects and suggested that IgG2 dominance results from immune responses to polysaccharide antigens such as lipopolysaccharide (LPS). When LPS binds to toll-like receptors (TLRs), it triggers the release of tumor necrosis factor-alpha (TNF-α), which activates dendritic cells. This activation increases CD4^+^ T cell activity and promotes IgG2 production [50]. Notably, several cardiovascular disorders, including CHD, are associated with elevated TNF-α levels (Fig. 1).

Chronic *H. pylori* infection contributes to atherosclerosis by promoting systemic inflammation through cytokines such as TNF-α, interleukin-6 (IL-6), and interleukin-1 beta (IL-1β). These inflammatory mediators impair endothelial function and accelerate plaque formation, in part by increasing levels of fibrinogen, thrombin, and intercellular adhesion molecules [51,52]. This inflammatory cascade activates macrophages and T lymphocytes, leading to proliferation of smooth muscle cells and extracellular matrix, which has a significant role in the development of atherosclerosis as one of the major risk factors for CVDs. Nevertheless, a large population-based study found no evidence of an association between *H. pylori* and inflammatory cytokines [53]. Although the aforementioned mechanism remains unconfirmed, Slomiany et al. have proposed an alternative molecular pathway [54,55]. In their model, LPS from the *H. pylori* cell wall activates mitogen-activated protein kinase (MAPK), c-Jun N-terminal kinase (JNK), and p38 kinase via toll-like receptor 4 (TLR4). This activation leads to increased expression of nitric oxide synthase (NOS) and cyclooxygenase-2 (COX-2) genes. Furthermore, *H. pylori* enhances COX-2 expression, resulting in higher production of thromboxane A2 (TXA2), a potent stimulator of platelet aggregation [56], and elevated levels of prostaglandins such as PGE2 [55]. Activation of the MAPK pathway also stimulates the epidermal growth factor receptor (EGFR), a key regulator of cell proliferation and apoptosis (Fig. 1) [55]. However, further research is needed to determine whether *H. pylori* acts as a causative agent or merely accelerates the progression of the disease. Additionally, *H. pylori* modulates lipid metabolism. Studies have shown that the infection inhibits lipoprotein lipase through cytokines such as TNF-α and increases the production of free radicals, increasing in triglycerides (TG) and a decrease in HDL-C. Free radicals also oxidize low-density lipoprotein cholesterol (LDL-C), promoting atherosclerosis, and enhance the chemotaxis of platelets and leukocytes, which lead to thrombosis [[57], [58], [59]]. The next remarkable mechanism is the similarity of *H. pylori* and human proteins. According to Sulewska et al., structural similarities between *H. pylori* surface proteins and heat shock protein 60 (HSP60) on endothelial cells may trigger cross-reactive immune responses and causing local inflammation of the artery [60].

## 4 H. pylori and metabolic dysfunction-associated steatotic liver disease (MASLD)

### 4.1 Prevalence of MASLD and gender differences

Researchers estimate that MASLD accounts for the majority of chronic liver diseases worldwide and recognize it as a major global health threat, resulting in substantial research and treatment expenditures by governments [71]. The prevalence of MASLD in the world is about 25 %, varying among different regions, with the highest prevalence in the Middle East (32 %), South America (31 %), Asia (27.37 %), North America (24.13 %), and Europe (23.71 %) [72].

In 2023, Dahiya et al. reported an increase in mortality among peptic ulcer disease (PUD) hospitalizations associated with MASLD in the United States between 2009 and 2019, compared to PUD cases without MASLD. Although hospitalized MASLD patients with PUD had more comorbidities, they experienced lower mortality rates, shorter hospital stays, and reduced overall healthcare costs. The study also showed that patients with MASLD-related PUD were more likely to receive upper endoscopy than those without MASLD. Notably, the authors stated that the NIS database covers 97 % of the U.S. population, making their findings broadly representative of MASLD-related PUD hospitalizations [73].

Gender significantly modifies the relationship between *H. pylori* infection and MASLD. Wang et al. (2021) found that *H. pylori* infection increases MASLD risk specifically in women [74].

The relationship between MASLD and *H. pylori* has long remained controversial due to inconsistent findings across studies. For example, a 2018 study by Fan et al. examined 21,456 individuals and reported different results, suggesting that the relationship is not consistent across all populations. The study showed that although univariate analysis suggested a positive association between *H. pylori* infection and MASLD, this link lost significance after adjustment for body mass index (BMI) and blood pressure. These results suggest that BMI mediates the observed association between *H. pylori* and MASLD [27]. Recent meta-analyses, including a comprehensive study of over 230,000 individuals predominantly of Asian ethnicity, have demonstrated a modest but statistically significant association between *H. pylori* infection and both prevalent and incident MASLD [75]. However, heterogeneity among studies arises from differences in population demographics, diagnostic methods for both *H. pylori* and MASLD, and adjustment for confounding factors such as BMI and metabolic syndrome components. Mechanistically, persistent *H. pylori* infection promotes MASLD progression through chronic systemic inflammation, IR, and gut microbiome alterations that increase hepatic inflammation and fibrosis risk [76].

### 4.2 Insulin resistance and metabolic dysfunction

Researchers initially proposed IR as one of the primary mechanisms linking *H. pylori* infection to MASLD [77]. Several studies have explored how *H. pylori* infection influences MASLD through its effect on IR. For instance, a 2012 study by Polyzos et al. examined 28 patients with MASLD and found that they were more likely to be infected with *H. pylori* compared to the control group. The study suggested that this effect is mediated through an increase in IR. According to the findings, *H. pylori*-infected patients exhibited higher levels of glucose, insulin, and homeostatic model assessment for insulin resistance (HOMA-IR) compared to individuals with no history of H. pylori infection [78].

### 4.3 Inflammation and cytokine activation

Chronic inflammation and abnormal signaling pathways can cause *H. pylori*-induced IR in MASLD [79]. In this context, *H. pylori*–associated inflammation characterized by elevated levels of C-reactive protein (CRP), TNF-α, and IL-6 impair hepatic insulin signaling and contribute to disease progression in MASLD [80]. These markers can activate kinases such as IKK/NF-kB and JNK, which can exacerbate IR via Ser-phosphorylation [81].

Inflammation is well recognized as a key contributor to MASLD pathogenesis [82]. Various inflammatory cytokines, including CRP, TNF-α, IL-6, and IL-1β, play a role in *H. pylori* infection [83,84]. Polyzos et al.'s study shows that individuals with positive *H. pylori* seropositivity exhibit higher levels of TNF-α and IR, despite having lower circulating adiponectin levels. *H. pylori* infection can elevate TNF-α levels, while adiponectin tends to increase as a compensatory response to the proinflammatory cascade. This process occurs either directly through *H. pylori* infection or indirectly through increased IR, with TNF-α contributing in both scenarios [78]. Also, according to Maleki et al.'s study, Hs-CRP is a reliable non-invasive biomarker for MASLD, with its levels being higher in patients with metabolic dysfunction-associated steatohepatitis (MASH) compared to those without MASH [85]. Additionally, Polyzos et al. demonstrated that MASLD patients with *H. pylori* infection had elevated levels of Hs-CRP [78]. The above evidence indicates that inflammation plays a key role in the relationship between *H. pylori* infection and MASLD. Through the JNK signaling pathway [86], this inflammatory process reduces liver glycogen levels, leading to decreased glucose gene expression and accelerated lipolysis [81].

*H. pylori* infection also modulates lipid metabolism, a mechanism that contributes to MASLD. Studies have shown that leptin, which is elevated in individuals with MASLD, can play a role in hepatic steatosis [87,88]. Additionally, studies have shown that *H. pylori* infection can influence leptin levels. For instance, one study revealed that *H. pylori* infection negatively affects serum leptin levels [89]. In contrast, Nishi et al. found that *H. pylori* infection increases leptin levels [90]. In fact, leptin can reduce VLDL-C and fat deposition in liver tissue by inhibiting stearoyl-CoA desaturase [91], and it can also impair insulin action by phosphorylating serine-318 [92].

Changes in lipid profiles influence the relationship between MASLD and *H. pylori*. One of the key characteristics of MASLD is the presence of fat deposits in the liver [93]. The study by Kotronen et al. found that insulin clearance is related to liver fat content [94], suggesting that IR, which mediates both MASLD and *H. pylori*, also impact fat deposition in the liver. Furthermore, increased liver fat can worsen IR, creating a vicious cycle where liver cells become saturated with TG and free fatty acids (FFA) [95] also, a study showed that MASLD patients have elevated levels of HOMA-IR, TC, TG, and LDL-C along with low HDL-C. The study also found that eradicating *H. pylori* can improve IR and correct lipid abnormalities [96]. Summary of the studies that have investigated the relationship between MASLD and *H. pylori* infection is provided in Table 2. While several studies report a positive association between *H. pylori* infection and MASLD, others find no significant relationship. Several methodological and population-related factors account for these conflicting findings. Differences in study populations including ethnicity, age distribution, and prevalence of metabolic risk factors can influence both the prevalence of *H. pylori* infection and susceptibility to MASLD [97]. Additionally, differences in diagnostic criteria for MASLD, ranging from imaging to histological confirmation, influence case definitions and reduce comparability across studies [98]. Researchers also use different methods to detect *H. pylori* infection, with some studies relying on serological tests that cannot distinguish active from past infection, while others use more specific tests such as urea breath or histology [71]. Furthermore, analytical approaches differ in controlling for confounding factors such as BMI, diabetes status, and lifestyle variables, which can modulate the observed associations. These factors collectively contribute to heterogeneity in findings and underscore the need for standardized methodologies and well-controlled longitudinal studies to clarify the true nature of the relationship between *H. pylori* and MASLD.

**Table 2:** Summary of studies on the relationship between MASLD and *H. pylori* Infection.

Author (Country)	population (n)	Key Findings	Refs.
Jamali et al. (Iran)	100	*H. pylori* eradication had no effect on liver/metabolic parameters	[104]
Okushin et al. (Japan)	13737	No association with MASLD	[105]
Sumida et al. (Japan)	130	Infection may contribute to MASH progression	[106]
Ki Baeg et al. (Korea)	3663	No association with MASLD	[107]
Joo Kang et al. (United states)	5404	Increased MASLD prevalence in infected individuals	[108]
Cai et al. (China)	2051	No association with MASLD	[109]
Yu Y et al. (China)	20389	Significant association with MASLD	[109]
Nengguang Fan et al. (China)	28,171	No independent association after adjusting for BMI	[110]
Chen et al. (China)	91	The presence of *H. pylori* may be an independent risk factor for MASLD	[111]
Ifard et al. (Iran)	130	*H. pylori* infection in MASLD patients is not clinically significant	[112]
Abo-Amer et al. (Egypt)	646	*H. pylori* is an independent MASLD risk factor	[113]
Alvarez et al.(Guatemala)	424	*Helicobacter* species, including *H. pylori*, did not show any link to MASLD CagA/VacA strains linked to MASLD	[114]
Siddiqui et al. (Pakistan)	399	MASLD develops earlier among *H. pylori*-infected dyspeptic patients in the 30–50 age group	[115]
Han et al. (Japan)	1784	*H. pylori* does not show a significant link with CAP-defined MASLD	[116]

*H. pylori*: *Helicobacter pylori*, MASLD: Metabolic dysfunction-associated steatotic liver disease, LFC: liver fat content, LFT: liver function tests, CAP: controlled attenuation parameter, MASH: metabolic dysfunction-associated steatohepatitis.

### 4.4 Gut microbiota and intestinal permeability

There is growing evidence linking microbiomes to diseases like MASLD in humans. Researchers initially based this evidence on mouse studies [23], and later extended it to include humans [99,100]. A retrospective study on gut immune function and the mucosal barrier found that MASLD significantly increases intestinal permeability. Furthermore, studies have shown that dysbiosis of the gut microbiota and subsequent disruption of the mucosal barrier can exacerbate MASLD by interfering with gut-liver communication [100]. An additional retrospective study involving 57 patients with biopsy-proven MASLD concluded that the histological types of MASLD that lead to liver complications and mortality are influenced by intestinal dysbiosis and alterations in the metabolic function of the gut microbiome [101]. According to available evidence, *H. pylori* causes dysbiosis of the intestinal microbiota, which is linked to MASLD, as *H. pylori* damages the intestinal mucosa, exacerbates intestinal permeability, and facilitates the entry of bacterial endotoxins into the liver via the portal vein, leading to increased inflammation [102,103].

However, establishing the existence or non-existence of a relationship between *H. pylori* infection and MASLD is complex and controversial. More studies, especially cohort studies, are needed to determine the effect of *H. pylori* treatment or eradication on the clinical course of MASLD. Additionally, researchers need to conduct further experimental studies to uncover the mechanisms linking the two, as both diseases have high prevalence. Clarifying their relationship helps accelerate the recovery process.

## 5 The relationship between H. pylori infection and diabetes mellitus (DM)

### 5.1 Epidemiological evidence and study variability

DM describes a group of metabolic diseases defined by hyperglycemia, due to impaired insulin function or secretion [117]. The global rise in diabetes has become one of the most critical national concerns. According to the International Diabetes Federation, there are 425 million diabetes patients worldwide, also WHO states that 90 % of diabetes patients have T2DM [118]. Some endocrine diseases, including thyroid autoimmune diseases, diabetes, and primary hyperparathyroidism, are related to *H. Pylori* infection, and *H. Pylori* infection is prevalent among diabetics however, the etiology of *H. Pylori* infection in diabetic patients remains unclear [119].

Simon et al. first investigated the relationship between *H. pylori* and DM in 1989 [120]. Since then, numerous studies have explored this connection. For example, Zhou et al. studied 1288 individuals. They found a positive association between *H. pylori* infection and T2DM, which may be further exacerbated when combined with traditional risk factors such as age, hypertension, and obesity. It was found that individuals aged 60 or older with *H. pylori* infection were at a greater risk of T2DM compared to those younger than 50 and without the infection [19].

There are potential confounders, such as age, gender, and BMI, that can influence the relationship between *H. pylori* and T2DM. Additionally, most studies have used cross-sectional designs rather than cohort designs, which limits causal interpretations. Another issue is that many studies rely solely on serological tests to detect *H. pylori*, which is less useful as a clinical diagnostic method due to its low specificity. Pyo et al. addressed these issues by adjusting for potential confounding metabolic and lifestyle factors in their study. Additionally, they conducted a longitudinal study instead of a cross-sectional one, and they used both serological and histological tests to assess *H. pylori* status. However, even after accounting for these factors, past *H. pylori* infections did not show a correlation with diabetes, impaired glucose tolerance (IGT), diabetic nephropathy, or poor glycemic control [121].

In the next study conducted by Cheng et al., the relationship between *H. pylori* and one of the most important factors related to T2DM, i.e., blood glucose levels, was investigated. According to the results, glycemic levels did not differ between patients with active *H. pylori* infection and those without it. However, the "active infection" group required significantly more glycemic treatment than the "non-active infection" group. In fact, an increased intensity of glycemic therapy (especially sulfonamides) resulted in comparable A1C levels in patients with active *H. pylori* infection [18].

Recent evidence also points to a significant association between *H. pylori* infection and glycemic control in diabetes. A 2024 study demonstrated significant associations between *H. pylori* infection, diabetes duration, and HbA1c levels, suggesting that chronic infection worsen glycemic dysregulation in affected individuals [20]. A summary of studies investigating the relationship between *H. pylori* and diabetes risk is provided in Table 3. The relationship between *H. pylori* infection and T2DM remains controversial, with conflicting evidence primarily due to differences in study design, population characteristics, and infection assessment methods. Cross-sectional studies frequently report higher prevalence of *H. pylori* infection among diabetics, but longitudinal data are less consistent regarding causality. Confounding factors such as obesity and metabolic syndrome complicate interpretation. Proposed mechanisms include *H. pylori*-induced chronic inflammation, oxidative stress, and hormonal dysregulation (e.g., altered ghrelin and leptin levels), which impair insulin sensitivity and glycemic control, particularly in genetically predisposed individuals. This complex interplay underscores the need for further well-designed prospective studies to clarify the causal pathways.

**Table 3:** Summary of studies on the relationship between DM and *H. pylori* Infection.

Author(country)	population(n)	Key Findings	Refs.
Oluyemi et al. (Nigeria)	200	No increased *H. pylori* infection in T_2_DM patients	[150]
Jafarzadeh et al. (Iran)	200	No link between CagA-positive strains and T2DM	[151]
Vafaeimanesh et al. (Iran)	429	*H. pylori* Infection increases insulin resistance in T2DM patients	[119]
Hsieh et al. (Taiwan)	2070	Chronic infection linked to higher HbA1c and reduced insulin secretion	[152]
Bonfigli et al. (Italy)	154	*H. pylori* Eradication improved glucose control in T2DM by reducing inflammation	[153]
Nam et al. (South Korea)	119	*H. pylori* eradication is not influenced by diabetes	[154]
Kouitcheu et al.(Cameroon)	954	*H. pylori* infection is more common in DM patients and raises HbA1c levels	[155]
Ghasemi et al. (Iran)	99	Similar *H. pylori* infection rates and IgG levels in diabetic and non-diabetic patients.	[156]
Alzahrani et al. (Saudi Arabia)	421	No association between *H. pylori* and diabetes	[157]
Kim et al. (South Korea)	66706	No increase in mortality after *H. pylori* treatment in T2DM	[158]
Kim et al. (Korea)	5181	Eradication did not increase mortality in T2DM patients	[158]
Kim et al. (Korea)	124	Eradication reduced HbA1c in men <65 with T2DM or pre-DM	[159]

*H. pylori*: *Helicobacter pylori*, T_2_DM: Type 2 diabetes mellitus, DM: Diabetes mellitus, ، HbAc1: Glycated hemoglobin.

### 5.2 Role of insulin resistance

One mechanism linking *H. pylori* and diabetes is IR. The physiological concept of IR refers to a reduced response to high levels of insulin in target tissues, which can exacerbate disorders such as metabolic syndrome, MASLD, and T2DM [122]. A systematic review showed that *H. pylori* and IR are related independently of confounding factors [123]. Also, a 2009 study found that *H. pylori* significantly increases IR in 1107 individuals [124]. Contrary to previous studies, Park et al. reported that there is no change in metabolic and inflammatory parameters such as IR and CRP after eradication of *H. pylori* [125]. Studies have indicated that IR can be triggered by inflammation [126] or hormonal changes [127], and perhaps *H. pylori* can trigger IR by causing chronic inflammation or affecting hormones, especially digestive hormones [128].

### 5.3 Hormonal changes: ghrelin and leptin

Another mechanism mentioned is the connection between *H. pylori* and diabetes through hormones. Various studies have demonstrated that gastritis caused by *H. pylori* infection can affect the secretion of hormones such as leptin and ghrelin [129,130]. These two hormones are effective in energy homeostasis [131]. In addition, these hormones can affect obesity and glucose homeostasis [132,133] ghrelin itself can cause weight gain [134], whereas leptin, which adipocytes produce, increases energy expenditure [135]. *H.pylori* infection increases ghrelin secretion, promoting weight gain and contributing to the development of diabetes [130]. *H. pylori* infection can also enhance leptin secretion [90], and leptin hormone also contribute to the development of IR and, consequently, T2DM [136]. Also a study by Rahman et al. demonstrated that inflammation and oxidative stress can damage insulin molecules, and therefore inflammation caused by *H. pylori* infection can impair insulin production (Fig. 1) [137]**.**

### 5.4 Inflammatory markers and cytokines

The first stage of infection is characterized by polymorph nuclear cells, but as the infection becomes chronic, mononuclear cells replace these cells. In people infected with *H. pylori*, mononuclear cells can produce several cytokines that can affect other tissues and organs, increasing extra intestinal diseases [138]. A 2005 study highlighted that, in response to *H. pylori* infection, the host immune system releases pro-inflammatory factors such as CRP [139], IL-6, and TNF-α [140], which are involved in IR and DM (Fig. 1) [141]. Although researchers have not fully understood the exact relationship between CRP levels and the development of diabetes, numerous studies have demonstrated a significant correlation between elevated CRP levels and DM [[142], [143], [144]]. Also, Sprenger et al. reported in a retrospective study that high levels of IL-6 play a role in T2DM [145]. In addition to the role of the previous two inflammatory markers, researchers have suggested that the increase in TNF-α production in adipose tissue plays a key role in inducing peripheral IR [146].

While no study has conclusively demonstrated a link between *H. pylori* and T2DM, it cannot be ignored that the pathophysiology of T2DM is complex, and lifestyle, daily activity level, dyslipidemia, etc., as well as *H. pylori* play a significant role. Therefore, larger prospective studies are needed to investigate the effect of *H. pylori* infection on diabetes. Further interventional studies will also be useful in studying the benefits of treating and eradicating *H. pylori* infection on T2DM clinical outcomes.

### 5.5 Emerging molecular mechanisms

Recent molecular studies have significantly advanced our understanding of the specific pathways linking *H. pylori* infection to T2DM and metabolic dysfunction. Bioinformatics and transcriptomic analyses have identified five key hub genes including TLR4, ITGAM (Integrin alpha M), C5AR1 (Complement component 5a receptor 1), FCER1G (High affinity immunoglobulin epsilon receptor subunit gamma), and FCGR2A (Fc gamma receptor IIA) that are upregulated in both *H. pylori* infection and T2DM, suggesting these genes mediate shared inflammatory and immune pathways implicated in disease progression [147,148]. Notably, researchers have identified activation of the NLRP3 inflammasome has emerged as a central mechanistic link. The CagA virulence factor of *H. pylori* can directly stimulate NLRP3 inflammasome activation, resulting in increased secretion of pro-inflammatory cytokines such as IL-1β and IL-18, which promote systemic inflammation, IR, and β-cell dysfunction [149]. In addition, recent research has elucidated the role of the c-Jun/miR-203/SOCS3 signaling pathway in hepatocytes: *H. pylori* infection activates c-Jun, leading to upregulation of miR-203, which in turn suppresses SOCS3 (a negative regulator of insulin signaling). This cascade disrupts normal insulin signal transduction and further contributes to IR [148]. Collectively, these findings highlight the molecular complexity of *H. pylori's* role in metabolic disease and underscore the importance of targeting these pathways in future therapeutic strategies.

In summary, the interplay between chronic inflammation, IR, hormonal changes, and emerging molecular pathways provides a plausible biological basis for the observed epidemiological associations between *H. pylori* infection and T2DM. These mechanisms act synergistically, with persistent infection triggering systemic inflammation and metabolic disturbances that promote the onset and progression of diabetes in susceptible individuals. This integrated perspective highlights the importance of future research that combines epidemiological, clinical, and molecular approaches to fully elucidate the role of *H. pylori* in metabolic disease.

## 6 H. pylori, gastric microbiota, and microbiome-mediated mechanisms in chronic disease

*H. pylori* infection now plays a recognized role as a major modulator of the gastric microbiome. In *H. pylori*-infected individuals, the bacterium often becomes the dominant species in the stomach, resulting in a marked reduction in overall microbial diversity and a decrease in the abundance of beneficial commensal genera such as *Roseburia*, *Streptococcus*, and *Lactobacillus* [160,161]. This decline in diversity appears more pronounced in individuals infected with virulent CagA-positive strains and alters the relative abundance of key bacterial phyla, including a reduction in *Actinobacteria*, *Bacteroidetes*, and *Firmicutes*, and an increase in *Proteobacteria* and *Haemophilus* [161,162]. The mechanisms underlying these shifts include *H. pylori's* production of urease, which raises gastric pH and alters the stomach's ecological niche, enabling its own persistence while suppressing other microbes [161]. Chronic infection also affects gastric physiology, including acid secretion and mucin structure, further shaping the microbial environment [163]. As a result, *H. pylori*-associated dysbiosis is not only characterized by reduced alpha diversity (species richness) but also by distinct changes in community structure (beta diversity), which contribute to mucosal inflammation and disease progression [164]. Importantly, successful eradication of *H. pylori* typically achieved through antibiotic-based therapies leads to a substantial decrease in the abundance of *H. pylori* and a gradual restoration of gastric microbial diversity. After eradication, the relative abundance of non-*H. pylori* commensals, including *Proteobacteria* and other beneficial taxa, increases, although some changes in community composition persist for months or longer [162]. Beyond the stomach, *H. pylori* infection and its treatment can also impact the gut microbiota. Antibiotic therapy causes transient gut dysbiosis, reducing beneficial genera and potentially affecting immune and metabolic homeostasis [162]. These microbiome alterations increasingly appear to contribute in the pathogenesis of extra-gastric diseases, including cardiovascular disease, MASLD, and T2DM. Mechanistically, *H. pylori*-induced dysbiosis can enhance mucosal inflammation, disrupt barrier function, and promote systemic immune activation, all of which are recognized contributors to chronic metabolic and cardiovascular conditions [162]. In summary, *H. pylori* infection profoundly alters the gastric microbiome, reducing microbial diversity and promoting dysbiosis. These changes, along with shifts in the gut microbiota following eradication therapy, play a key role in the development and progression of chronic diseases through immune, metabolic, and inflammatory pathways.

## 7 Conclusion

This review synthesizes growing evidence linking *H. pylori* infection to CVD, MASLD, and T2DM. Across all three conditions, chronic systemic inflammation emerges as a common mechanistic theme. However, the strength of evidence and the implicated biological pathways vary notably across diseases. For CVD, the evidence is strongest and most consistent. Numerous cohort and meta-analysis studies support a causal or contributory role for *H. pylori*, particularly via mechanisms such as vascular inflammation, cytokine activation (e.g., TNF-α, IL-6), endothelial dysfunction, and dyslipidemia. There is also growing support for autoimmunity via molecular mimicry and prothrombotic pathways (e.g., TXA2 elevation). In the case of MASLD, the association with *H. pylori* is more controversial. Some studies suggest a link mediated by IR, chronic inflammation, and alterations in gut-liver axis signaling. However, several large-scale cohort studies have failed to confirm an independent relationship after adjusting for confounders like BMI. This inconsistency suggests that *H. pylori* may act as a co-factor rather than a primary driver in MASLD development. For T2DM, evidence indicates potential associations through inflammation, hormonal disruption (e.g., ghrelin, leptin), and microbiota-mediated pathways. However, most of these studies are cross-sectional and cannot determine causality. A few longitudinal studies offer more convincing evidence, especially regarding *H. pylori's* influence on glycemic control and treatment intensity. Still, further investigation is warranted.

Overall, while inflammation and metabolic disruption are recurring themes, CVD presents the most robust causal narrative, followed by a more tentative link in T2DM, and conflicting evidence in MASLD. Future studies should prioritize mechanistic research and randomized controlled trials that explore the differential impact of *H. pylori* eradication across various metabolic and vascular outcomes.

## CRediT authorship contribution statement

**Navid Maleki:** Writing – review & editing, Writing – original draft, Methodology. **Alireza Mohammadzadeh:** Writing – review & editing, Writing – original draft. **Jalal Mardaneh:** Writing – review & editing, Writing – original draft. **Hossein Pazoki:** Writing – review & editing, Writing – original draft. **Elyas Nattagh-Eshtivani:** Writing – review & editing, Writing – original draft, Supervision, Methodology, Investigation, Conceptualization.

## Clinical and public health implications

Given the high global prevalence of *H. pylori*, especially in low- and middle-income countries, the following strategies may offer public health and clinical benefit.

- Routine screening for *H. pylori* in individuals with unexplained cardiovascular risk factors, poor glycemic control, or metabolic syndrome, particularly in high-prevalence regions.
- Personalized eradication therapy based on genotypic resistance profiling, especially in patients with concurrent metabolic disorders.
- Integration of *H. pylori* management into NCD prevention programs, such as hypertension and diabetes screening campaigns.
- Public health education aimed at promoting hygiene and early childhood prevention to reduce transmission in endemic areas.
- Surveillance of antibiotic resistance patterns to guide empirical treatment protocols and reduce treatment failure.

These recommendations, if implemented alongside existing NCD control strategies, help reduce long-term disease burden. Ultimately, future research should prioritize longitudinal cohort studies and randomized controlled trials to better establish causal relationships between *H. pylori* infection and chronic diseases such as CVD, MASLD, and T2DM. Special attention should be given to high-prevalence regions, particularly in low- and middle-income countries, where genetic and environmental factors modulate disease outcomes. Mechanistic studies are also needed to explore the specific roles of *H. pylori* virulence factors like CagA, host genetic polymorphisms (e.g., TLR and cytokine genes), and microbiota-mediated pathways. Integrating multi-omics approaches, including metagenomics and transcriptomics, can further elucidate the complex host–microbe interactions and identify potential therapeutic targets.

## Data availability statement

Data sharing not applicable to this article as no datasets were generated or analyzed during the current study.

## Funding

The author(s) declare that no financial support was received for the research, authorship, and/or publication of this article.

## Declaration of competing interest

The authors declare no conflict of interest.
